# Fetal arrhythmia and heart disease during COVID-19 pandemic in patients who underwent echocardiography

**DOI:** 10.61622/rbgo/2026rbgo51

**Published:** 2026-09-23

**Authors:** Fernanda de Almeida Mattos, Gláucia Maria Penha Tavares, Gustavo Yano Callado, Alexandre Rodrigues Marra, Christiane Simioni, Renata Gomes Castello, Rita de Cássia Sanchez, Eduardo Félix Martins Santana

**Affiliations:** 1 Hospital Israelita Albert Einstein Faculdade Israelita de Ciências da Saúde Albert Einstein São Paulo SP Brazil Faculdade Israelita de Ciências da Saúde Albert Einstein, Hospital Israelita Albert Einstein, São Paulo, SP, Brazil.; 2 Hospital Israelita Albert Einstein São Paulo SP Brazil Hospital Israelita Albert Einstein, São Paulo, SP, Brazil.; 3 University of Iowa Health Care Department of Internal Medicine Iowa City Iowa USA Department of Internal Medicine, University of Iowa Health Care, Iowa City, Iowa, USA.; 4 Universidade Federal de São Paulo Department of Obstetrics São Paulo SP Brazil Department of Obstetrics, Universidade Federal de São Paulo, São Paulo, SP, Brazil.; 5 Stanford University School of Medicine Division of Infectious Diseases & Geographic Medicine Stanford CA USA Division of Infectious Diseases & Geographic Medicine, Stanford University School of Medicine, Stanford, CA, USA.

**Keywords:** Fetal arrhythmia, Echocardiography, Heart diseases, Congenital heart diseases, COVID-19, Pandemics, Comorbidity, Tertiary Care Centers, Prevalence, Risks, Brazil

## Abstract

**Objective:**

To evaluate the characteristics and the occurrence of fetal heart diseases and arrhythmias in pregnancies that underwent fetal echocardiography examination during COVID-19 pandemic.

**Methods:**

A retrospective cohort study was conducted using data extracted from the Fetal Ultrasound and Fetal Medicine Departments of Hospital Israelita Albert Einstein, a tertiary hospital in São Paulo, Brazil, from January to December 2021. The sample was analyzed using chi-square or Fisher's exact tests, and Student's t-test or the Mann-Whitney test. Logistic regression was performed to assess the risk of fetal arrhythmia and/or heart disease.

**Results:**

A total of 148 fetuses were included in the analysis. The prevalence of fetal heart disease/arrhythmia was higher among patients with maternal comorbidities (73.3% vs. 39.1%), fetal defects (14.3% vs. 0.8%), and functional abnormalities (26.7% vs. 4.5%). Maternal COVID-19 showed no significant association with fetal cardiac outcomes. Maternal comorbidity, fetal malformation, and the presence of functional abnormalities were identified as independent risk factors.

**Conclusion:**

Maternal comorbidities were associated with fetal arrhythmia and/or heart disease, but no causal relationship was found between COVID-19 and adverse fetal cardiac outcome.

## Introduction

Congenital heart diseases (CHDs) in neonates are severe conditions that often necessitate multiple early-life surgical interventions and are associated with significant long-term health burdens. These complex medical needs can adversely affect the quality of life of affected individuals.^(1)^ The American Heart Association and the American Academy of Pediatrics have recognized that individuals with CHDs are at increased risk for lifelong morbidities, further emphasizing the chronic nature of these conditions.^(1)^

Recent studies have demonstrated that COVID-19 can induce myocardial injury and left ventricular dysfunction in pregnant women, with a reported mortality rate of 13.3%, primarily due to malignant arrhythmias occurring during hospitalization.^(2)^ These findings point toward a possible link between COVID-19 and cardiovascular compromise.

Preliminary evidence has begun to explore the impact of maternal COVID-19 on fetal cardiac health. One retrospective cohort study found a notable increase in fetal arrhythmia diagnoses—particularly supraventricular tachycardia and complete heart block—during the pandemic period, without concurrent structural heart defects.^(3)^ Another investigation reported a sharp rise in the incidence of fetal left-right (LR) asymmetry disorders during the post-pandemic period in China, with maternal COVID-19 in the first trimester identified as a significant risk factor. Strikingly, over 90% of the associated anomalies in these cases involved cardiac malformations.^(4)^

This study aimed to clarify whether maternal COVID-19 increases the risk of fetal arrhythmia or CHD, while accounting for established risk factors like comorbidities and fetal malformations.

## Methods

This was a retrospective cohort study carried out at the *Hospital Israelita Albert Einstein* (HIAE), a tertiary hospital in São Paulo, Brazil. Data were extracted from the printed report systems of the fetal echocardiography department within the Fetal Medicine sector at HIAE and from questionnaires attached to the reports, which contained patient-provided information to assist physicians during the examinations.

The study included all patients who underwent fetal echocardiography at HIAE between January and December 2021. Patients were eligible if complete data were available in both the printed report and the pre-examination questionnaire administered by the hospital's department. Patients with incomplete information in either the report or the questionnaire were excluded.

Data on COVID-19 were self-reported by the patients through the questionnaire. Patients who reported infection specified the type of diagnostic test performed. However, among those who denied infection, there were both women with a negative reverse-transcriptase polymerase chain reaction (RT-PCR) test result and women who were not tested during pregnancy.

The study evaluated the following variables: COVID-19, gestational age at the time of infection, maternal age, presence of obstetric pathologies, amniotic fluid abnormalities, estimated fetal weight and percentile, presence of fetal defects, fetal echocardiogram findings, presence of fetal heart disease and arrhythmias, and types of heart diseases and arrhythmias. It was not possible to determine whether patients who underwent RT-PCR testing were symptomatic at the time of testing, as this information was not included in the medical records.

The sample was initially described using means, standard deviations, minimum and maximum values, medians, and quartiles for quantitative variables, and absolute and relative frequencies for qualitative variables.

The association between variables of interest and outcomes was evaluated using chi-square or Fisher's exact tests, and Student's t-test or the Mann-Whitney test, depending on the distribution characteristics. Data normality was assessed using the Shapiro-Wilk test, boxplots, histograms, and quantile-quantile plots. Logistic regression was applied to assess the risk of fetal arrhythmia and/or heart disease, considering the variables of interest as covariates.

According to Peduzzi's recommendation, a multivariable logistic regression model should include no more than one explanatory variable per 10 events in the sample.^(5)^ Given the sample's event count and these guidelines, interpreting risk factors using a multivariable model was not advisable. Therefore, risk estimates were calculated for each individual variable.

All analyses were performed using the Statistical Package for the Social Sciences (SPSS), version 26.0, with a significance level of 5%.

The project was approved by the Research Ethics Committee of Hospital Israelita Albert Einstein, São Paulo, Brazil, under opinion number 5.934.543, CAAE number 62037022.1.0000.0071, and institutional Research Project Management System (SGPP) number 5251-22. The Ethics Committee approved the exemption from the Informed Consent Form (ICF), as the study exclusively analyzed data from the institutional database without collecting patient-identifying information or conducting any additional procedures on participants or their biological samples.

## Results

A total of 304 patients underwent fetal echocardiography at HIAE between January and December 2021. However, only 148 cases had available data on fetal arrhythmia and/or heart disease and had self-reported information about COVID-19 infection and could be included in the analysis. Each fetus was analyzed individually, including those from multiple pregnancies, with each treated as a separate case. In instances where a mother had multiple clinical visits, only data from the most recent visit were considered. The sample included 148 patients, with a mean and median gestational age of 27 weeks, ranging from 16 to 39 weeks. The mean maternal age was 34.5 years, with a standard deviation of 4.3 years, ranging from 22 to 44 years. The majority of pregnancies were singleton (93.2%), and 11.6% resulted from in vitro fertilization. Regarding pre-existing maternal conditions, 42.6% of patients had at least one chronic disease, with hypothyroidism (50.8%) and thrombophilia (20.6%) being the most prevalent. Obstetric pathologies were identified in 16.4% of the sample. Among these cases, 45.8% had gestational diabetes mellitus (GDM), while 54.2% had other conditions, which included urinary tract infections during pregnancy, first-trimester bleeding, pregnancy-induced hypertension (without preeclampsia), anemia, and low-lying placenta. These conditions were grouped together due to their low prevalence within the sample. In the sample, 23.0% of patients tested positive for COVID-19 during pregnancy, with an average gestational age at the time of infection of 12 weeks. Of the total sample, 15 (10.1%) fetuses were diagnosed with either arrhythmia or congenital heart disease. Among these, three (20.0%) had arrhythmias and 12 (80.0%) had congenital heart defects. The three cases of arrhythmia were classified as extrasystoles: two (66.7%) as atrial extrasystoles and one (33.3%) as ventricular extrasystole. Among congenital heart defects, acyanotic conditions—specifically ventricular septal defects and complete atrioventricular septal defects—were the most prevalent, accounting for 7 (77.8%) cases. Additionally, 10 fetuses (6.8%) exhibited functional cardiac abnormalities, with the right chamber (28.6%) dilation and pericardial effusion (42.9%) being the most common findings. A detailed description of the sample is provided in table 1.

**Table 1:** Sample characterization

Characterization		Mean (SD)
Maternal age		34.5(4.3)
Gestational age (completes weeks)		27.7(3.4)
Gestational age at COVID-19 diagnosis (completed weeks)		12.6(11.6)
**Variables**		**n(%)**
Maternal disease presence		63(42.6)
Type of maternal disease		
	Chronic hypertension	6(9.5)
	Diabetes mellitus	1(1.6)
	Autoimmune disease	8(12.7)
	Thrombophilia	13(20.6)
	Cardiopathy	1(1.6)
	Hypothyroidism	32(50.8)
	Arrhythmias	1(1.6)
	Hyperthyroidism	0(0)
	Others	8(12.7)
Single pregnancy		138(93.2)
Fertilization (n=147)		17(11.6)
Obstetric pathology (n=146)		24(16.4)
Type of obstetric pathology		
	Pre-eclampsia	0(0)
	GDM	11(45.8)
	Congenital infections	0(0)
	Others	13(54.2)
Growth restriction (n=147)		
	<p10	1(0.7)
	<p3	0(0)
Fetal malformation (n=147)		3(2)
Type of abnormality (n=3)		
	T 21	1(33.3)
	T 18	0(0)
	T 13	0(0)
	Turner Syndrome	0(0)
	Others	2(66.7)
Fetal echocardiography abnormalities		22(14.9)
Fetal cardiopathy		12(8.1)
Type of cardiopathy (n=9)		
	Cyanotic	1(11.1)
	Acyanotic	7(77.8)
	Semilunar valve stenosis	1(11.1)
	Aortic arch anomalies	0(0)
	Univentricular physiology	0(0)
	Left ventricular hypertrophy	0(0)
Presence of functional abnormalities (n=147)		10(6.8)
Type of functional abnormalities (n=7)		
	Right chamber dilation	2(28.6)
	Pericardial effusion	3(42.9)
	Ventricular dysfunction	0(0)
	Tricuspid regurgitation	1(14.3)
	Other valves regurgitation	0(0)
	Others	1(14.3)
Presence of fetal arrhythmia		3(2)
COVID-19		34(23)
Fetal heart disease and/or arrhythmia		15(10.1)

GDM: gestational diabetes mellitus.

The study's primary outcome was the presence of fetal arrhythmia and/or congenital heart disease, observed in 15 (10.1%) participants. Table 2 details the association between maternal and gestational characteristics and this outcome. The prevalence of congenital heart disease and/or fetal arrhythmia was higher among patients with maternal comorbidity (73.3% vs. 39.1%, p=0.011), in pregnancies with fetal defects (14.3% vs. 0.8%, p=0.024), and when functional abnormalities were present (26.7% vs. 4.5%, p=0.001). COVID-19 was not associated with fetal arrhythmia and/or congenital heart disease, and no statistically significant differences were observed for the other characteristics (p>0.05). Table 2 presents the simple logistic regression models for the risk of fetal arrhythmia and/or congenital heart disease. Risk factors associated with fetal arrhythmia and/or congenital heart disease included maternal comorbidity (p=0.017), fetal malformation (p=0.014), and the presence of functional abnormalities (p=0.050). Specifically, patients with maternal diseases had a 4.3-fold increased risk compared to those without, while fetuses with malformations were 22 times more likely to develop arrhythmia and/or congenital heart disease. The presence of functional abnormalities increased the risk by 7.6 times. Other variables did not show statistically significant associations (p-values>0.05). Additionally, odds ratios for singleton pregnancy and obstetric pathology could not be estimated due to low frequencies in the group with arrhythmia and/or congenital heart disease.

**Table 2:** Comparison of the presence of fetal heart disease or arrhythmia by characteristics

Variables		Had fetal heart disease and/or Arrhythmia		p-value
		No 133 n(%)	Yes 15 n(%)	
Maternal age, mean (SD)		34.4(4.4)	35.6(3.2)	0.301***
Maternal disease presence		52(39.1)	11(73.3)	0.011*
Type of maternal disease				
	Chronic hypertension	4(3.0)	2(13.3)	0.280**
	Diabetes mellitus	1(0.7)	0(0)	>0.999**
	Autoimmune disease	7(5.3)	1(6.6)	0.692*
	Thrombophilia	10(7.5)	3(20.0)	0.549*
	Hypothyroidism	26(19.5)	6(40.0)	0.784*
Gestational age (completed weeks), mean (SD)		27.8(3.1)	27(4.9)	0.780****
Single pregnancy		123(92.5)	15(100)	0.271*
Underwent fertilization		16(12.0)	1(6.6)	0.531*
Obstetric pathology		24(18.0)	0(0)	0.081*
Fetal abnormality		1(0.7)	2(13.3)	0.024**
Fetal echocardiography abnormalities		7(5.3)	15(100)	<0.001*
Presence of functional abnormalities		6(4.5)	4(26.6)	0.001*
COVID-19		33(24.8)	1(6.6)	0.113*

* Ch-square test: n (%).

** Fisher's exat test: n (%).

*** Student's T-test: Mean (standard deviation).

**** Mann-Whitney test: Median [quartiles]. Bold: values with statistically significant relevance to the study. n=148.

**Table 3:** Logistic regression for fetal arrhythmia and/or heart disease

Variables		Odds ratio (OR) (IC 95%)	p-value
Maternal age		1.07 (0.94; 1.23)	0.300
Maternal disease presence			
	Yes	4.28 (1.30; 14.17)	0.017
	No	Reference	-
Single pregnancy		-	0.999
Gestational age (completed weeks)		0.92 (0.78; 1.10)	0.363
Underwent fertilization			
	Yes	0.52 (0.06; 4.21)	0.538
	No	Reference	-
Obstetric pathology		-	0.998
Fetal abnormality			
	Yes	22 (1.86; 260.65)	0.014
	No	Reference	-
Fetal echocardiography abnormalities		-	0.995
Presence of functional abnormalities			
	Yes	7.64 (1.87; 31.19)	0.005
	No	Reference	-
COVID-19			
	Positive	0.22 (0.03; 1.71)	0.147
	Negative	Reference	-

odds ratios for singleton pregnancy and obstetric pathology could not be estimated due to low frequencies in the group with arrhythmia and/or congenital heart disease

## Discussion

This retrospective cohort study conducted at a tertiary hospital in São Paulo, Brazil, analyzed 148 pregnancies with available data on fetal echocardiography and maternal COVID-19 status. The study found that 10.1% of fetuses were diagnosed with either arrhythmia or CHD, with arrhythmias accounting for 20% and CHDs for 80% of these cases. The most prevalent CHDs were acyanotic lesions, specifically ventricular septal defects and complete atrioventricular septal defects. Functional cardiac abnormalities were observed in 6.8% of fetuses, with right chamber dilation and pericardial effusion being the most common. Statistical analysis revealed significant associations between the presence of fetal arrhythmia and/or CHD and maternal comorbidity, fetal malformations, and functional abnormalities. However, no significant association was found between maternal COVID-19 and the occurrence of fetal arrhythmia or CHD.

It is noteworthy that the mean gestational age at COVID-19 diagnosis in our cohort was 12.6 weeks, indicating that most infections we could include in the analysis occurred during the first/second trimester. It is possible that earlier gestational exposure to COVID-19 may have a different pathophysiological impact compared to infections acquired later in pregnancy, when fetal cardiovascular development is more advanced.^(6,7)^

Some studies have explored the impact of maternal COVID-19 on fetal cardiac outcomes. A recent study observed an increased incidence of fetal arrhythmias, particularly supraventricular tachycardia and complete heart block, during the COVID-19 pandemic, without concurrent structural heart defects.^(3)^ Another investigation reported a significant rise in fetal left-right asymmetry disorders during the post-pandemic period in China, with maternal COVID-19 in the first trimester identified as a significant risk factor.^(8)^ Notably, over 90% of the associated anomalies in these cases involved cardiac malformations. Additionally, a study presented evidence linking maternal COVID-19 during early pregnancy to fetal situs inversus, a condition often associated with CHDs.^(9)^ Furthermore, a systematic review highlighted that COVID-19 during pregnancy can be associated with cardiovascular complications in both the mother and fetus, including arrhythmias and structural heart defects.^(10)^

Fetal cardiovascular findings across studies included various types of arrhythmias, especially fetal tachycardia and bradycardia, with reported rates ranging from 1% to 20%.^(10)^ Additionally, Doppler studies and fetal echocardiography revealed altered blood flow dynamics, such as increased pulsatility and resistance indices in the umbilical and uterine arteries, as well as decreased fetal cardiac output in some cases of severe maternal disease.^(11)^ Some case reports and small cohort studies also described instances of fetal myocarditis, cardiomyopathy, and even fetal death related to cardiovascular complications, including hydrops fetalis in the context of significant cardiac involvement.^(12)^

These findings were supported by several individual studies. Mercedes et al. reported that 20% of fetuses from COVID-19-positive mothers with myocardial injury developed fetal bradycardia.^(2)^ Sinaci et al. documented tachycardia in 5.4% and bradycardia in 1.3% of 224 COVID-19-positive pregnant women.^(13)^ Kato et al. and Gubbari et al. described cases of fetal tachycardia and myocarditis, the latter associated with non-immune hydrops and a reduced ejection fraction of 35%.^(12,14)^ Furthermore, Doppler assessments in studies by Anuk et al. and Sule et al. demonstrated increased resistance and pulsatility indices, as well as prolonged pulmonary artery acceleration time, suggesting subtle signs of fetal hemodynamic stress in pregnancies affected by maternal COVID-19.^(11,15)^ Although not always reaching statistical significance, these observations indicate a trend toward impaired placental or fetal cardiovascular function in the setting of maternal COVID-19.

The potential impact of maternal COVID-19 on fetal cardiovascular development may involve several pathophysiological mechanisms. One proposed mechanism is the direct viral invasion of placental tissues, leading to placental inflammation and dysfunction, which can compromise fetal oxygen and nutrient supply. Studies have reported significant pathological changes in the placental tissue of COVID-19 positive mothers, including vascular malperfusion and inflammatory lesions. Another mechanism involves the maternal immune response to infection, characterized by a cytokine storm that can lead to systemic inflammation and endothelial dysfunction, potentially affecting fetal development. Furthermore, the hypercoagulable state induced by COVID-19 may result in microthrombi formation in placental vessels, further impairing placental function. These mechanisms underscore the importance of monitoring pregnant women with COVID-19 for potential adverse fetal outcomes, even in the absence of direct vertical transmission of the virus.

Importantly, maternal comorbidities emerged as the most significant associators of fetal arrhythmia and CHD in our study. Conditions such as hypothyroidism and thrombophilia, which were prevalent in our cohort, may independently alter fetal development through mechanisms including altered placental perfusion, systemic inflammation, or hormonal imbalances. This finding highlights the critical role of maternal health status in fetal cardiac development, potentially outweighing the influence of maternal COVID-19 in determining risk.

Several limitations of this study must be acknowledged. The retrospective design inherently limits the ability to establish causal relationships and may have resulted in underreporting of asymptomatic or mild COVID-19 cases. The sample size, while adequate for detecting moderate to large effects, may have been underpowered to identify smaller but clinically relevant associations, particularly when examining outcomes by trimester of infection. Another limitation is the lack of information regarding maternal symptomatology at the time of RT-PCR testing. It remains unclear whether infected patients were asymptomatic or symptomatic, which could have implications for fetal outcomes, as symptomatic infection—particularly with systemic manifestations—may pose a greater risk to fetal development. Importantly, data on COVID-19 vaccination status were not available for this cohort, which represents an additional limitation given the potential impact of vaccination on maternal and fetal outcomes. Additionally, the single-center nature of the study and the predominance of a tertiary care population may limit generalizability, as the cohort may have been enriched for pregnancies with pre-existing risk factors. The study period in 2021 primarily captured infections with the Delta variant, and the findings may not be fully applicable to other COVID-19 strains with differing pathogenic profiles. Furthermore, while standardized echocardiography protocols were employed, the detection of subtle functional abnormalities may have varied depending on operator expertise and timing of assessment.

Despite these limitations, the study provides valuable insights into fetal cardiac outcomes during the COVID-19 pandemic. The absence of a significant association between maternal infection and fetal arrhythmias or CHD suggests that traditional risk factors remain the primary predictors of these conditions. However, continued surveillance is warranted as new variants emerge and longer-term follow-up data become available. Future research should prioritize prospective, multicenter studies with detailed phenotyping of maternal infection characteristics, including variant type, and immune response, as well as standardized longitudinal fetal cardiac assessments. Integration of placental pathology and biomarkers of inflammation may further elucidate potential mechanisms linking maternal infection to fetal cardiovascular outcomes.^(16)^

## Conclusion

In conclusion, maternal comorbidities and fetal malformations were significant risk factors for fetal arrhythmia/CHD, but COVID-19 during pregnancy did not independently increase risk. These findings reassure patients and clinicians, though ongoing surveillance is warranted as new variants emerge. Prenatal screening should prioritize traditional risk factors while remaining vigilant for rare COVID-19-related complications.

## Data Availability

The research data are described in the article presented.
